# Ultra-short echo time MR imaging in assessing cartilage endplate damage and relationship between its lesion and disc degeneration for chronic low back pain patients

**DOI:** 10.1186/s12880-023-01014-5

**Published:** 2023-04-20

**Authors:** Zhilin Ji, Yue Li, Weiqiang Dou, Yaru Zhu, Yin Shi, Yuefen Zou

**Affiliations:** 1 Department of Radiology, Tianjin Hospital, Jiefangnan Road, Hexi District, Tianjin, 300211 P.R. China; 2 Department of Radiology, The First Affiliated Hospital of Nanjing Medical University, Nanjing, 210000 P.R. China; 3 GE Healthcare, MR Research China, Beijing, P.R. China

**Keywords:** Chronic low back pain, Disc degeneration, Ultra-short echo time, Magnetic resonance imaging, Cartilage endplate, Bony vertebral endplate, Pfirrmann grade, Total endplate score

## Abstract

**Objective:**

To investigate the feasibility of ultra-short echo time (UTE) magnetic resonance imaging (MRI) in the assessment of cartilage endplate (CEP) damage and further evaluate the relationship between total endplate score (TEPS) and lumbar intervertebral disc (IVD) degeneration for chronic low back pain patients.

**Materials and methods:**

IVD were measured in 35 patients using UTE imaging at 3T MR. Subtracted UTE images between short and long TEs were obtained to depict anatomy of CEP. The signal-to-noise ratio (SNR) and contrast-to-noise ratio (CNR) were calculated to assess the image quality quantitatively. A new grading criterion for endplate damage evaluation was developed based on Rajasekaran.S grading system in this study. Two radiologists were employed to evaluate CEP and bony vertebral endplates (VEP) using this new grading criterion and assess TEPS, independently. Cohen’s kappa analysis was applied to evaluate the inter-observer agreement of endplate damage assessment between two radiologists, and the Kendall’s TAU-B analysis was employed to determine the relationship between TEPS and IVD degeneration evaluated with Pfirrmann grading.

**Results:**

Well structural CEP was depicted on subtracted UTE images and confirmed by high SNR (33.06±2.92) and CNR values (9.4±2.08). Qualified subtracted UTE images were used by two radiologists to evaluate the degree of CEP and VEP damage. Excellent inter-observer agreement was confirmed by high value in Cohen’s kappa test (0.839, P < 0.001). Ensured by this, 138 endplates from 69 IVDs of 35 patients were classified into six grades based on the new grading criterion and TEPS of each endplate was calculated. In addition, the degeneration degree of IVDs were classified into five grades. Finally, using Kendall’s TAU-B analysis, significant relationship was obtained between endplate damage related TEPS and IVD degeneration (r = 0.864, P < 0.001).

**Conclusion:**

Ensured by high image quality, UTE imaging might be considered an effective tool to assess CEP damage. Additionally, further calculated TEPS has shown strong positive association with IVD degeneration, suggesting that the severity of endplate damage is highly linked with the degree of IVD degeneration.

## Introduction

Up to 85% of people have chronic low back pain in their lifetime, severely affecting their life and work [1]. There are many reasons reported for chronic low back pain, including trauma, metabolic diseases, benign and malignant tumors and of particular intervertebral disc (IVD) degeneration [2, 3].

Lumbar IVD includes central nucleus pulposus, peripheral annulus fibrosus, and superior and inferior cartilaginous endplates (CEP) anatomically. Among these components, CEP as the most fragile structure is considered one important reason for IVD degeneration, if lesion occurs [4]. CEP functions as equalizing loading between IVD and vertebral body, and maintains normal hydrostatic pressure within the nucleus. It plays an important role in transporting nutrient between IVD and vertebral body [5, 6]. The lesions of CEP, including thinning, defects and calcification, may prevent the passage of nutrients from IVD and result in a progress of IVD degeneration.

Magnetic resonance imaging (MRI) has been proposed for lumbar spine imaging because of well structural description of IVD with superior tissue contrast but without radiation risk [7]. Using conventional T1-weighted MRI, Rajasekaran. S et al. classified endplates into six grades according to severity of damage (Table 1). With the proposed Rajasekaran grading system, a so-called Total Endplate Score (TEPS) by summing endplate defect scores over both rostral and caudal endplates of disc was further calculated to help for clinical treatment [8]. Although the whole endplate was well evaluated with Rajasekaran grading system, CEP and bony vertebral endplates (VEP), both of which are essential components of endplate, was however not able to be distinguished for clinical evaluation, largely due to low signal and tissue contrast on conventional T1w images. Accurate evaluation of CEP damage could thus not be achieved. Chen. KC et al. considered that VEP lesions may be related to structural or biomechanical changes of the adjacent CEPs. The manifestations of CEP, i.e., focal thinning, focal defect and biomechanical weakening, may lead to the vulnerability of VEP [9]. Therefore, it is necessary to distinguish between CEP and VEP in order to observe this pathological process. Structural damage of the CEP, allowing for the IVD to bulge into the vertebral body, altering the distribution of disc loading, leading to nucleus pulposus and annulus fibrosus mechanical perturbations and aberrant disc cell metabolism, which may ultimately affect disc health. [1]. So far, in clinic no gold standard is available to evaluate CEP damage. Although conventional MRI allows to visualize VEP changes non-invasively [9], CEP is not able to be imaged properly largely due to fast signal decay by ultra-short T2 relaxation time. Well structural depiction of both tissues with sufficient image contrast thus remains challenging so far [10].

Ultra-short Echo Time (UTE) MR imaging, as a promising technique for high signal-to-noise ratio (SNR), has been applied to assess CEP [1, 11, 12]. With flexible short or long echo time selection, UTE images dominated with bound & free water protons or free water protons can be acquired, respectively [13]. Bound water weighted UTE images can thus be easily obtained by subtracting UTE images from short to long TEs to obtain well depicted CEP structure (1,14). Moreover, CEP and VEP can be distinguished clearly. With this advantage, it was assumed that UTE images could have the potential to accurately evaluate CEP and VEP damage. However, as far as we know, no relevant study has been explored till now.

Therefore, this study mainly aimed to investigate the feasibility of UTE imaging in evaluating the CEP and VEP damage for chronic low back pain patients. Based on Rajasekaran grading system, a modified grading criterion with additional definition of a new grading was applied for grading endplate damage on UTE images. Corresponding TEPS index was calculated for each endplate. In addition, the relationship of TEPS was further investigated with lumbar IVD degeneration determined by clinically applied Pfirrmann score system [15] to reflect the severity of damaged CEP.

## Materials and methods

This prospective study was approved by the review board of The First Affiliated Hospital of Nanjing Medical University. All participants gave informed consent.

### Subjects

Thirty-five patients (18 males, 17 females; mean: 42±16 years) that had clinically suspected lumbar IVD degenerative diseases, including those who experienced chronic or recurrent low back pain for at least 3 months with or without leg pain, were recruited from October 2020 to April 2021 in this study. Patients were excluded if they (1) were unable to tolerate long scan time due to severe low back pain or claustrophobia; (2) had history of lumbar spinal surgery or radiotherapy; (3) had spinal deformity, trauma; or benign and malignant vertebral tumors; (4) had hematologic diseases and other systemic diseases.

### MRI experiment

All MRI examinations were performed on a 3T MRI (Discovery 750w, GE Healthcare, USA) equipped with an eight-channel received only large flexible array coil. The imaging protocol included T2-weighted 2D fat-suppressed (FS) sequence, T1-weighted 2D fast spin-echo (FSE) sequence, T2-weighted 2D FSE sequence and a 3D gradient echo based UTE imaging sequence.

T2-weighted 2D FS imaging was applied for L1-S2 at sagittal view with field of view of 256 mm×256 mm. Other scan parameters comprised of TE = 85ms, TR = 2000ms, matrix size = 256 × 256, slice thickness = 2.0 mm, number of slices = 15, NEX = 2 and bandwidth = 41.67Mhz. Scan time was 1 min.

T1-weighted 2D FSE imaging was applied for L1-S2 at sagittal view with field of view of 320 mm×320 mm. Other scan parameters comprised of TE = 85ms, TR = 472ms, matrix size = 320 × 320, slice thickness = 4.0 mm, number of slices = 15, bandwidth = 62.50Mhz.

T2-weighted 2D FSE imaging was applied for L1-S2 at sagittal view with field of view of 320 mm×320 mm, Other scan parameters comprised of TE = 120ms, TR = 2588ms, matrix size = 320 × 320, slice thickness = 4.0 mm, number of slices = 15, bandwidth = 31.25Mhz.

3D double-TE UTE imaging, using a non-selective RF excitation hard pulse combined with 3D radial acquisition [14], was applied for L2-S1 with field of view of 360 mm×360 mm at sagittal view. The first short echo of 0.03ms detected signals from both long and short T2 components that dominated with bound & free water protons. The second long echo of 6ms measured signals from longer T2 components which were considered with free water protons only. Bound water protons dominated UTE images could thus be easily obtained by subtracting the second echo from the first echo. Other scan parameters consisted of TR = 12.5ms, matrix size = 360 × 360, slice thickness = 4.0 mm, number of slices = 20, NEX = 1, flip angle = 15° and bandwidth = 83.33Mhz. Scan time was 2 min 48s.

### Grading of endplate defects and IVD degeneration and TEPS calculation

On the basis of six-level Rajasekaran grading [8], a modified six-level grading system with extra diagnostic criteria for CEP and VEP damage was proposed in this study, including:

Grade I was normal CEP and VEP with good continuity, no Modic change; Grade II showed thinning CEP in part or whole but with good continuity, no interruption or defect, and intact VEP, no Modic change; Grade III diagnosed with CEP local depression, complete continuity and VEP was not involved, no Modic change; Grade IV showed CEP begin with defects, the continuity of CEP was broken with less than 25% of damage area and VEP involved, Modic change occurred; Grade V with the CEP damage area less than 50% of the total area and involved VEP, accompanied with Modic change; Grade VI mean that the damage area of CEP is greater than 50% of the total area or whole CEP involved and VEP was involved, along with Modic change.

With this new grading system, the corresponding TEPS index for each IVD was further calculated by summing endplate defect scores of both rostral and caudal endplates in each functional spine unit.

Pfirrmann grading, widely applied in clinic, was used to evaluate the degeneration of IVD on T2-weighted FSE lumbar imaging [15]: according to severity from 1 (the structure of IVD is homogeneous with bright hyperintense white signal intensity and normal disc height) to 5 (the structure of IVD is inhomogeneous with a hypointense black signal intensity and no more detectable difference between the nucleus and annulus, and the disc space is collapsed).

Whether Modic change exists was assessed by two radiologists. Modic type 1 means decreased signal intensity on T1-WI and increased signal intensity on T2-WI. Modic type 2 means increased on T1-WI and iso/hyperintenseon T2-WI. Modic type 3 means decreased on both T1-WI and T2-WI. If there is no one above type, it is judged as no Modic changes.

### Data analysis

All acquired UTE images were analyzed in ADW4.6 workstation (GE Medical Systems).

**Fig. 1:**
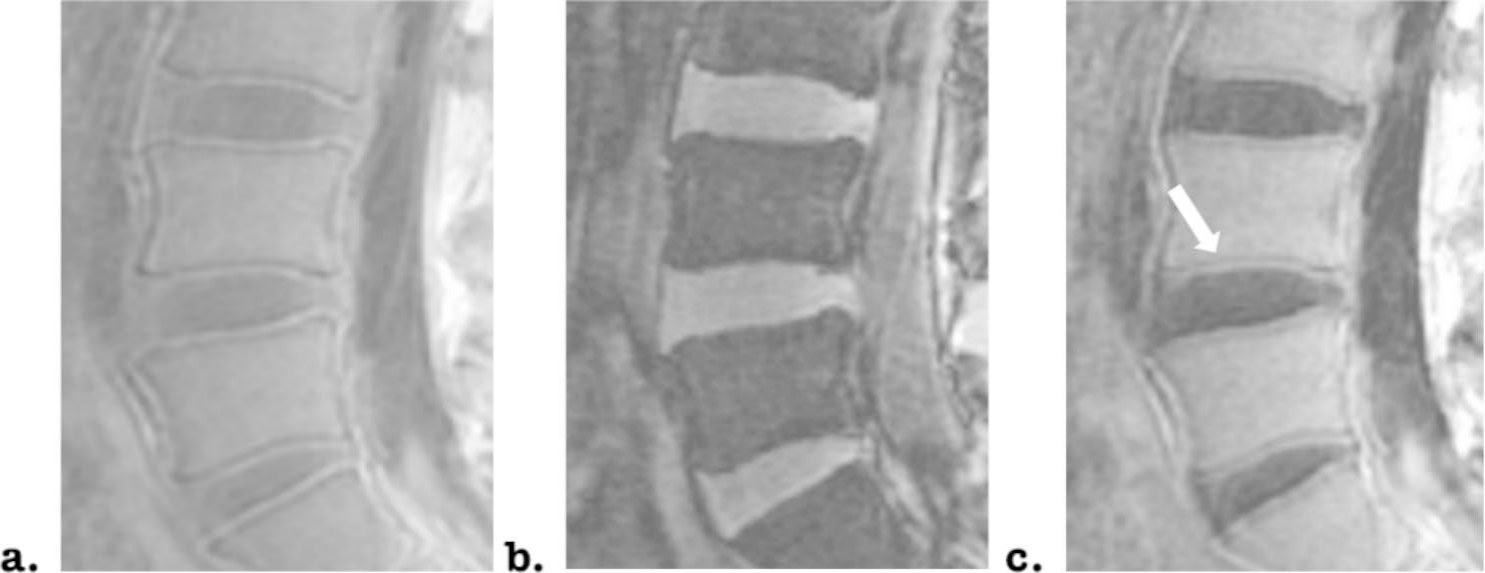
Subtracting a 3D ultrashort Echo Time (UTE) image acquired at short TE of 0.03 ms (a) by the one acquired at long TE of 6 ms (b), a resultant subtracted 3D UTE image (c) of a lumbar spine with relatively normal cartilage endplate (CEP) is presented at sagittal view. Well structural CEP (denoted by white arrow) is hyperintense as well as adjacent hypointense vertebral endplate. The effect of nucleus pulposus, annulus fibrosus was removed after image subtraction

**Fig. 2:**
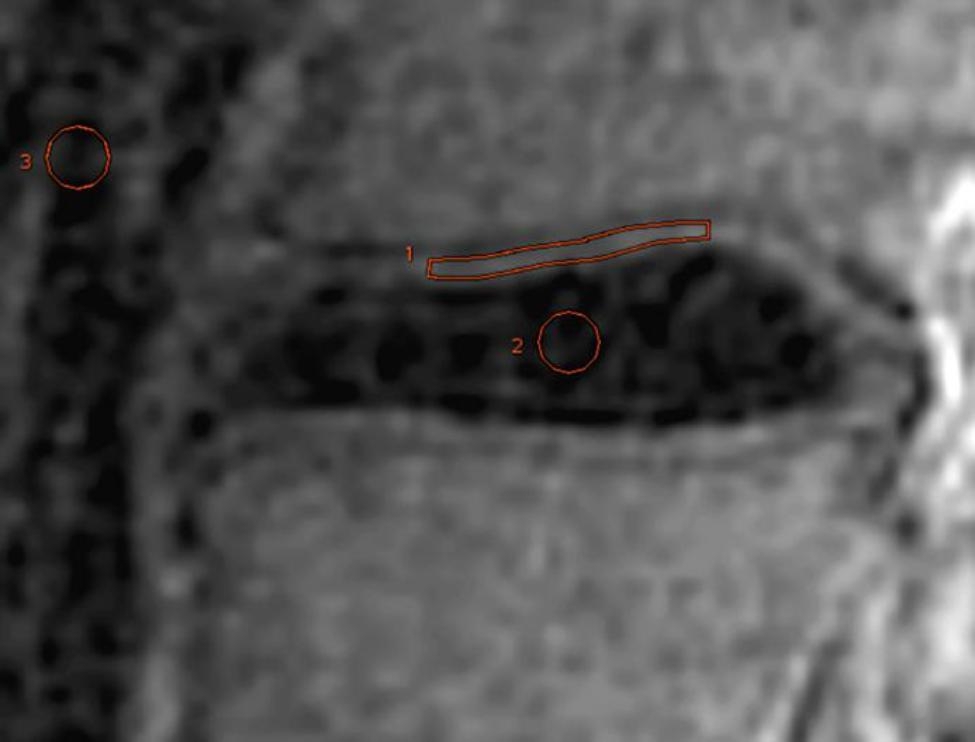
Representative images of ROIs in cartilaginous endplate (CEP), disc, and background in 25-year-old male volunteer. Mid sagittal image of subtracted ultra-short echo time images. ROI of CEP was drawn using freehand technique. In this image, ROI 1 is located on inferior CEP of L3 and 22 mm^2^ in area. ROI of disk (ROI 2) was circle and 8 mm^2^ in area. ROI of background (ROI 3) was circle and 9 mm^2^ in area

Bound water dominated UTE images of CEP were calculated with a subtraction of UTE images from short to long TEs (Fig. 1.a-c). The indexes of SNR and contrast-to-noise ratio (CNR) were calculated for the resultant subtracted UTE images to evaluate the image quality:

1 $$SN{R_x} = |{S_x} - {S_{background}}|/s$$

and

2 $$CN{R_{x - y}} = \left| {{S_x} - {S_y}} \right|/s$$

where S_x_, S_y_ and S_background_ represented the mean signal intensities of ROI x for the clearest area of CEP, ROI y for the maximum contrast area selected from adjacent CEP and background ROI, respectively, and s was the standard deviation of the background noise (Fig. 2).

Two radiologists (J.Z.L, Z.Y.F, with 3-year and 20-year experience in musculoskeletal radiology, respectively) were employed to assess the CEP damage on subtracted UTE images. The disagreements were resolved by discussion between two radiologists.

### Statistical analysis

All statistical analyses were performed using SPSS software (version 26.0.0; SPSS Inc., Chicago, IL). Cohen’s Kappa test was used to evaluate the inter-observer agreement in assessing the degree of CEP defect between two radiologists. Kendall’s TAU-B test was adopted to analyze the correlation between TEPS and IVDs. P < 0.05 was considered the threshold of statistical significance.

## Results

### Endplate analysis in UTE imaging

One hundred and thirty-eight endplates of 69 IVDs from 35 patients were clearly shown on subtracted UTE images (Fig. 1c). Moreover, the image quality was confirmed by high SNR and CNR (mean SNR: 31.48±2.73; mean CNR: 9.1±1.97).

With Cohen’s Kappa test, excellent inter-observer agreement of CEP and VEP damage diagnosis between two radiologists was confirmed by high value of 0.839 (95%CI:0.925 ~ 1.05; P < 0.001). Disagreed diagnoses were occurred in CEPs with normal VEPs in grade II and III. Whether there is CEP damage alone on the basis of normal VEP has become a contradiction. Two radiologists achieved consensus after discussion.

**Fig. 3:**
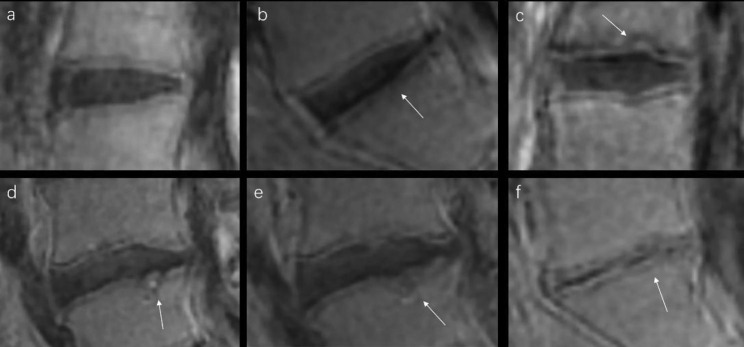
On the subtracted 3D UTE images, representative endplate images at each grade are shown. a: grade I, normal cartilage endplate (CEP) and bony vertebral endplate (VEP). b: grade II, CEP local thinning (denoted by white arrow). c: grade III, focal sunken of CEP (white arrow), complete continuity and VEP was not involved. d,e: grade IV and V, the defect of CEP and VEP can be observed (white arrow), adjacent to normal CEP and VEP. f: grade VI, CEP and VEP almost defected completely (white arrow), with obvious collapse of the disc

Ensured by reliable diagnosis, 138 endplates were assessed with a modified Rajasekaran grading system that developed in this study, which included 37 endplates for Grade I, 37 endplates for Grade II, 23 endplates for Grade III, 15 endplates for Grade IV, 12 endplates for Grade V, 14 endplates for Grade VI (Fig. 3.a-f). For total 69 discs of 35 patients enrolled in this study, 50% of CEP damages in L1-2 discs were grade 3, 41% of CEP damages in L2-3 discs were grade 3, 42% of CEP damages in L3-4 discs were grade 1, 30% of CEP damages in L4-5 discs were grade 2, 28% of CEP damages in L5-S1 discs were grade 2.

### Correlations between TEPS and IVD degeneration and demography

After scoring with the proposed new grading system, the corresponding TEPS for each IVD was further calculated by summing endplate scores of both rostral and caudal endplates in each functional spine unit. The resultant TEPS of total 69 IVDs was ranging from 2 scores to 12 scores, and distributed as follows: ten IVDs were evaluated with 2 scores, thirteen IVDs were with 3 scores, nine IVDs were with 4 scores, seven IVDs were with 5 scores, seven IVDs were with 6 scores, five IVDs were with 7 scores, eight IVDs were with 8 scores, two IVDs were with 9 scores, two IVDs were with 10 scores, one IVD was with 11 scores and five IVDs were with 12 scores.

IVD degeneration was evaluated based on Pfirrmann grading. Of total 69 IVDs, thirteen were Pfirrmann grade I, fifteen were Pfirrmann grade II, eighteen were Pfirrmann grade III, fourteen were Pfirrmann grade IV and nine were Pfirrmann grade V.

The distribution of discs with various degeneration degrees for each TEPS was shown in Table 2.

**Table 1:** Six level of endplate in Rajasekaran grading system

**Grade I**	Normal endplate, with no interruptions.	No Modic changes
**Grade II**	Thinning of the endplate, no obvious break.	
**Grade III**	Focal endplate defect with maintained endplate contour.	
**Grade IV**	Endplate defects < 25% of the endplate area.	Associated with Modic changes
**Grade V**	Endplate defects up to 50% of the endplate area.	
**Grade VI**	Extensive damaged endplate up to total destruction.	

**Table 2:** Incidence of various grades degeneration of intervertebral disc according to total endplate score (TEPS).

Pfirrmann grading	TEPS											
	2	3	4	5	6	7	8	9	10	11	12	Total
I	10	3										13
II		10	5									15
III			4	6	6	2						18
IV				1	1	3	8	1				14
V								1	2	1	5	9
Total	10	13	9	7	7	5	8	2	2	1	5	69

Using Kendall’s TAU-B analysis, significant relationship between the TEPS and IVD degeneration was revealed by high coefficient of 0.864 (P < 0.001).

Using Spearman correlation analysis, significant correlation was revealed between the TEPS and age with coefficient of 0.473 (P < 0.004). No significant association was however found between TEPS and gender.

## Discussion

Ensured by highly qualified UTE images, we for the first time investigated the feasibility of UTE imaging in assessing the damage of CEPs for patients with chronic low back pain. As showed in the results, the image quality of UTE imaging was confirmed by high SNR and CNR (mean SNR: 31.48 ± 2.73; mean CNR: 9.1 ± 1.97). Excellent inter-observer agreement of damaged CEP and VEP evaluation was confirmed by high value of 0.839 (95%CI: 0.925 ~ 1.05; P < 0.001) by Cohen’s kappa test.

Subtracted UTE imaging has been shown an effective clinical method to display CEP changes noninvasively, because it maintained bound water signal with ultrashort T2* and eliminated the effect of nucleus pulposus and annulus fibrosus by removing the signal contribution from free water protons [1, 14]. Based on this advantage, bound water weighted CEP presented hyperintense signal while the signal of VEP was hypointense. The structure of CEP could thus be well visualized and the image contrast between CEP and VEP was strong.

A modified CEP grading system, based on six-level Rajasekaran grading method [8], was developed in this study, defining additional criteria on CEP and VEP damage. Unlike displaying only the whole endplate on conventional T1 weighted imaging, UTE imaging with well distinguished CEP and VEP allows for accurate diagnosis of CEP and VEP damage. The original six-level Rajasekaran grading method without accurate grading for CEP and VEP is thus not sufficient, and a new grading system with extra new grading is highly requested in this study.

Chen. KC et al. considered that the defect of CEP might cause weakened or lost VEP bone mineral density, and then led to VEP damage [10]. In other words, the integrity of CEP ensured that VEP was not damaged. UTE imaging provided a possibility for observing pathological processes that transit from CEP damage to VEP damage in a noninvasive way. Damaged VEP appears mostly Schmorl’s nodule sign, a classic sign of endplate defect. When VEP defects, nucleus pulposus herniation presents as the characteristic Schmorl’s nodule and may be along with vertebral bone marrow involvement (e.g., edema), a so-called Modic change, which is also the boundary between grade III and IV. Grade III was defined as CEP with local depression but complete continuity, VEP was not involved and without Modic change. The corresponding cases in our scheme showed that the integrity of CEP ensured that VEP was not involved and vertebral bone marrow was not damaged on subtracted UTE images (Fig. 3c). Grade IV was defined as CEP begin with defects, the damaged area of CEP less than 25% of total area, VEP involved and along with Modic change, while the corresponding cases showed that both CEP and VEP were damaged with vertebral bone marrow involved on subtracted UTE images (Fig. 3d), so Modic change occurred. UTE imaging could display obviously the process grade III transforms to IV, which the damage extends from CEP to VEP. Subtracted UTE images could be observed that whether CEP and VEP damage, bone marrow involvement or not so that the corresponding TEPS could be calculated accurately for each IVD and used for further clinical application.

In addition, using Kendall’s TAU-B analysis, significant relationship between the TEPS and IVD degeneration was revealed by high coefficient of 0.864. It indicates that the increase of CEP degree might induce more serious disc degeneration, although pathologic results are requested for further validation. IVD degeneration was a major cause of chronic low back pain [2, 3].

Many factors contribute to disc degeneration including age and Body Mass Index (BMI). Rade. M et al. reported that when TEPS was included as predictor in multivariable model, the covariates such as age and BMI became insignificant that mean endplate defect was the prevalent factor in disc degeneration [16]. A minor interruption of endplate may be sufficient to cause significant changes in the mechanical environment of IVD and effect diffusion paces for maintaining IVD nutrition and nucleus pulpoid hydration [17, 18]. In addition, herniating annulus fibrosus may lead to CEP away from the subchondral bone and promoting endplate defect [19, 20]. In another way, anaerobic bacteria may also enter the IVD through the defect of endplate and cause IVD degeneration [21].

Rajasekaran. S et al. reported that the cut-off value of TEPS was 5, over which the prevalence of IVD degeneration was significantly higher. When TEPS is above 5, mechanical environment and nutritional pathways of IVD are blocked, IVD degeneration is impossible to reverse through natural healing or adaptive remodeling [4]. The following treatments of biological treatment, stem cell therapy, geneticmanipulation, anabolic stimuli, even growth factors that will be identified as possible treatments in the future were not suitable for these IVDs [8]. Therefore, it is necessary to evaluate TEPS accurately in order to help for the clinical treatment and evaluate disc degeneration grade. Subtracted UTE images could show well structural CEP images and the corresponding TEPS could be calculated accurately on UTE images.

This study also has some limitations. First, histologic references of patients corresponding to the new grades are lacked. Second, CEP minor structural damage (e.g., fissure or crack) may not appear in the UTE images, but these defects represent microscopic compositional changes, such as acidophilic degeneration, focal proliferation, calcification and cell death, which are known to occur in CEP [22]. Third, although Rade. M et al. mentioned that endplate defect was the prevalent factor in disc degeneration, the multi-aetiological nature and multifactorial condition (e.g., BMI, age, sex, genetics) of IVD degeneration may influence the assessment of CEP defects and their roles in IVD changes. Fourth, after long time scanning, some patients still showed motion artifacts due to low back pain. Finally, this study sample size was limited. Nonetheless, our study raises awareness that UTE imaging provides the opportunity to identify CEP defects which would be missed and reveals the diagnosis of CEP defects to be clinically beneficial in predicting the development and severity of IVD degeneration, which may have effects on clinical treatment options and patient outcomes.

## Conclusion

In conclusion, UTE imaging has been demonstrated promising in assessing CEP damage for patients with chronic low back pain. In addition, applying the new grading system, index of TEPS could be further calculated based on accurately classified endplates, and revealed close correlation with lumbar IVD degeneration evaluated by Pfirmann score system. With these findings, UTE imaging might be considered an effective tool in assessing CEP damage in clinic.

## Data Availability

The datasets analyzed in this study are available from the corresponding author on request.
